# Supplementary material for: Bacterial Infections and Their Cell Wall Ligands Differentially Modulate Doxorubicin Sensitivity in Triple-Negative Breast Cancer Cells
Source: Microorganisms. 2025 Oct 7;13(10):2317. doi: 10.3390/microorganisms13102317 (PMC12566298; doi:10.3390/microorganisms13102317)
Supplement: Supplementary file 1 [file microorganisms-13-02317-s001.zip › microorganisms-3904861-supplementary.pdf]

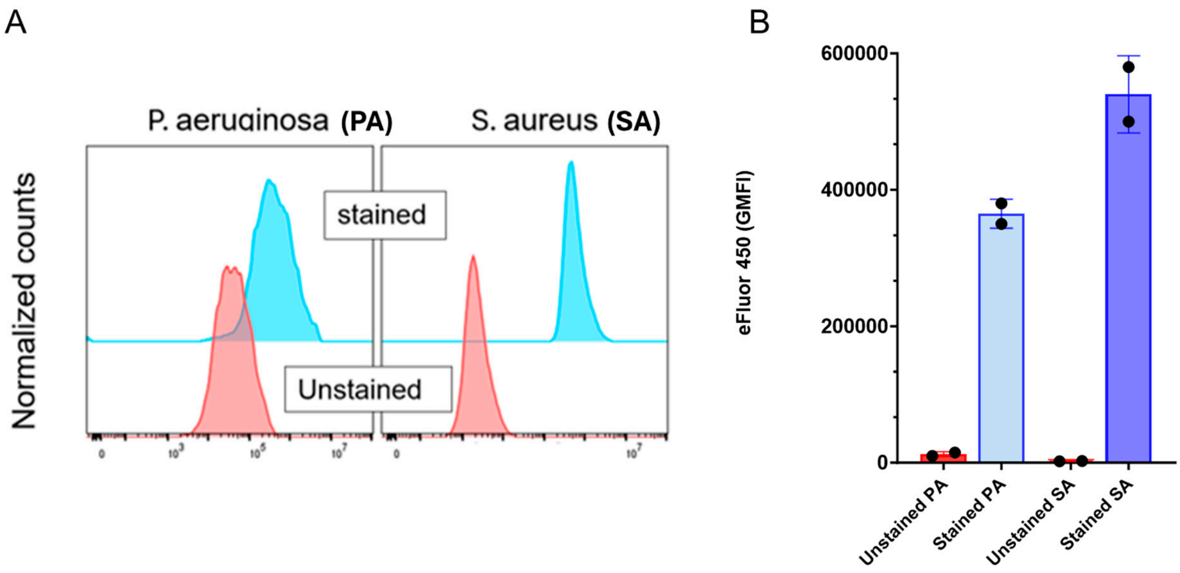

Figure S1: Comparison of *P. aeruginosa* and *S. aureus* staining using eFluor 450.
(A) Representative flow cytometry histograms showing eFluor 450 fluorescence intensity in *P. aeruginosa* (PA) and *S. aureus* (SA). Stained samples (blue) were compared to unstained controls (red) to assess background levels.
(B) Geometric mean fluorescence intensity (GMFI) of eFluor 450 in unstained and stained samples for *S. aureus* and *P. aeruginosa*. The results indicate more efficient labeling of *S. aureus* with eFluor 450 compared to *P. aeruginosa* (a Gram-negative bacterium), possibly due to interference from lipopolysaccharide in the outer membrane. This difference in bacterial labeling efficiency impacts the accurate quantification of the percentage of cells infected with *P. aeruginosa* versus *S. aureus*, limiting direct comparison between the two infection models.

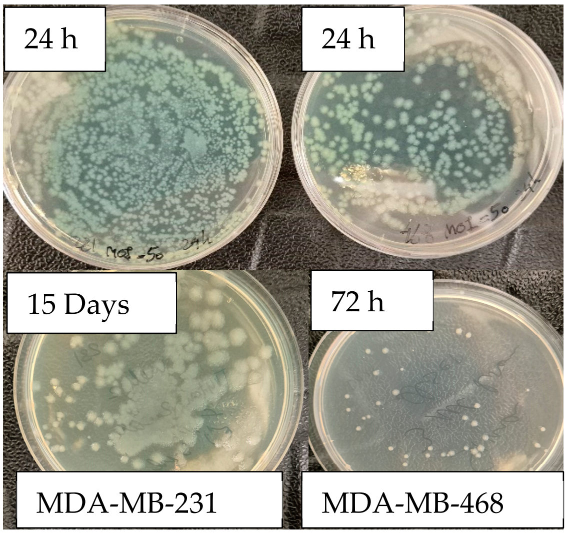

Figure S2: Viable intracellular *P. aeruginosa* recovered from MDA-MB-231 and MDA-MB-468 cells over time.
Cells were infected with *P. aeruginosa* at MOI 50, and viable intracellular bacteria were recovered by lysing 30,000 infected cells with Triton X-100. Lysates were plated on LB agar and incubated overnight at 37 °C. Colony formation was observed at 24 h and persisted for up to 15 days in MDA-MB-231 cells, indicating long-term intracellular survival. In contrast, *P. aeruginosa* was completely cleared from MDA-MB-468 cells by 3 days.

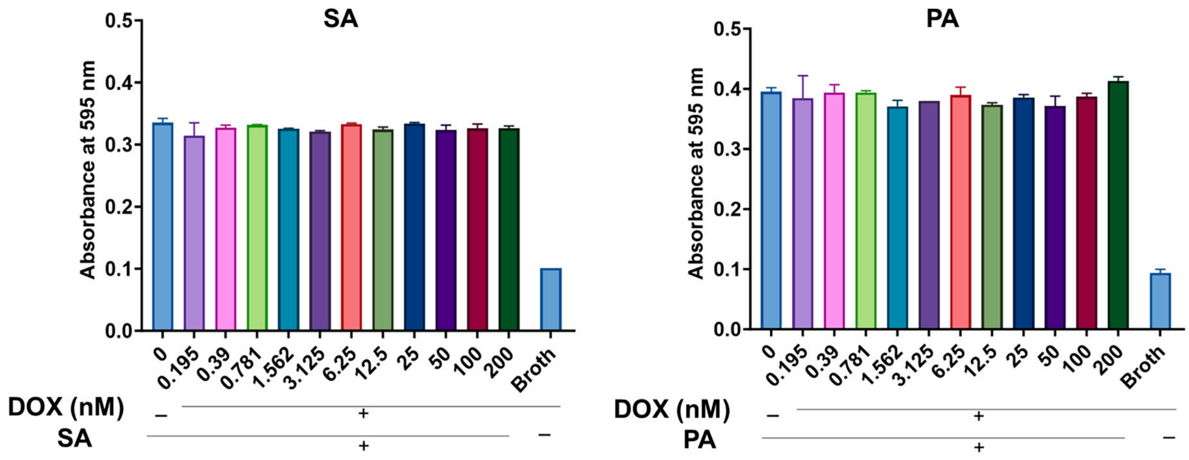

Figure S3: Doxorubicin does not affect the viability of *S. aureus* or *P. aeruginosa.* Bacterial cultures of *S. aureus* (SA) and *P. aeruginosa* (PA) were exposed to increasing concentrations of doxorubicin (0–200 nM) for 24 h. Viability was assessed by optical density measurements at 595 nm using a minimum inhibitory concentration (MIC) assay. No significant changes in absorbance were detected across doxorubicin concentrations, indicating that doxorubicin does not impair bacterial growth. “Broth” represents the negative control without bacterial inoculation. Data are presented as mean ± SD from three independent experiments.

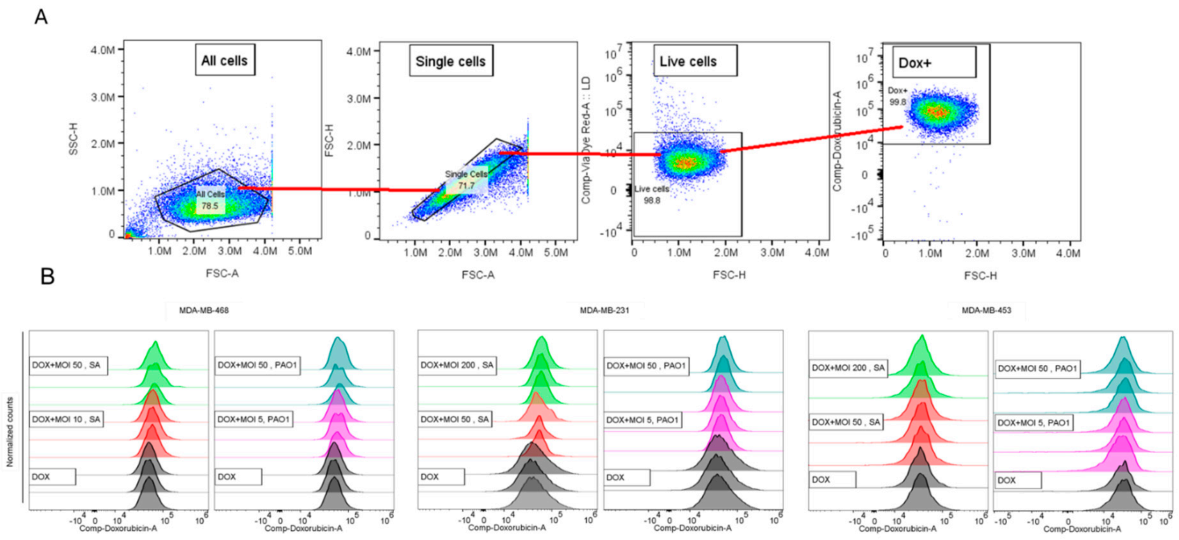

Figure S4. Flow cytometry strategy for analyzing intracellular doxorubicin accumulation in TNBC cell lines following bacterial infection.

A) Representative gating strategy. Cells were sequentially gated to exclude debris and select for single, viable cells. Doxorubicin-positive cells were then identified based on intrinsic doxorubicin fluorescence.

B) Overlay histograms show doxorubicin fluorescence intensity in MDA-MB-468, MDA-MB-231, and MDA-MB-453 cells under various treatment conditions. Cells were treated with 100 nM doxorubicin alone or in combination with *S. aureus* (SA) or *P. aeruginosa* (PA) at the indicated MOIs. Histograms represent normalized fluorescence count to visualize shifts in doxorubicin accumulation following bacterial infection.

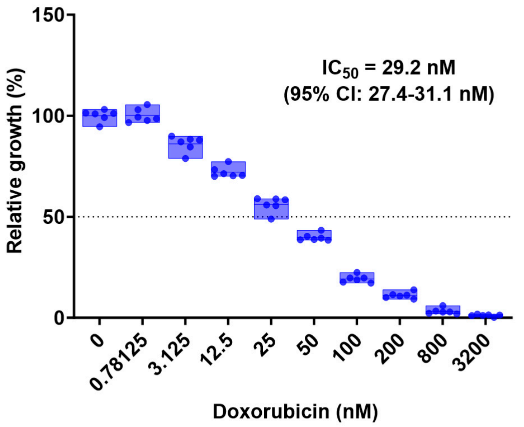

Figure S5: Determination of doxorubicin IC_{50} in MDA-MB-468 cells.
MDA-MB-468 cells were treated with increasing concentrations of doxorubicin (0–3200 nM) for 5 days. Relative growth was assessed using crystal violet staining and normalized to vehicle control (0 nM doxorubicin). The IC_{50} value was calculated to be 29.2 nM (95% CI: 27.4–31.1 nM), as indicated on the graph. Data represents the mean ± SD from at least six replicates.
